# Conformational Determinants of Phosphotyrosine Peptides Complexed with the Src SH2 Domain

**DOI:** 10.1371/journal.pone.0011215

**Published:** 2010-06-21

**Authors:** Joseph Nachman, Gerry Gish, Cristina Virag, Tony Pawson, Régis Pomès, Emil Pai

**Affiliations:** 1 Department of Biochemistry, University of Toronto, Toronto, Ontario, Canada; 2 Division of Molecular and Developmental Biology, Samuel Lunenfeld Research Institute, Mount Sinai Hospital, Toronto, Ontario, Canada; 3 Molecular Structure and Function, Hospital for Sick Children, Toronto, Ontario, Canada; 4 Departments of Medical Biophysics and Molecular Biology, University of Toronto, Toronto, Ontario, Canada; 5 Division of Genomics and Proteomics, Ontario Cancer Institute, Toronto, Ontario, Canada; University of Queensland, Australia

## Abstract

The inhibition of specific SH2 domain mediated protein-protein interactions as an effective chemotherapeutic approach in the treatment of diseases remains a challenge. That different conformations of peptide-ligands are preferred by different SH2 domains is an underappreciated observation from the structural analysis of phosphotyrosine peptide binding to SH2 domains that may aid in future drug design. To explore the nature of ligand binding, we use simulated annealing (SA) to sample the conformational space of phosphotyrosine-containing peptides complexed with the Src SH2 domain. While in good agreement with the crystallographic and NMR studies of high-affinity phosphopeptide-SH2 domain complexes, the results suggest that the structural basis for phopsphopeptide- Src SH2 interactions is more complex than the “two-pronged plug two-hole socket” model. A systematic study of peptides of type pYEEX, where pY is phosphotyrosine and X is a hydrophobic residue, indicates that these peptides can assume two conformations, one extended and one helical, representing the balance between the interaction of residue X with the hydrophobic hole on the surface of the Src SH2 domain, and its contribution to the inherent tendency of the two glutamic acids to form an α-helix. In contrast, a β-turn conformation, almost identical to that observed in the crystal structure of pYVNV bound to the Grb2 SH2 domain, predominates for pYXNX peptides, even in the presence of isoleucine at the third position. While peptide binding affinities, as measured by fluorescence polarization, correlate with the relative proportion of extended peptide conformation, these results suggest a model where all three residues C-terminal to the phosphotyrosine determine the conformation of the bound phosphopeptide. The information obtained in this work can be used in the design of specific SH2 domain inhibitors.

## Introduction

SH2 (Src homology 2) domains are found as modules in many proteins involved in cell signal transduction pathways. They bind to short stretches of amino acids that contain phosphorylated tyrosine residues (pTyr) and thereby mediate the interactions between proteins during cell signaling. Since these processes control cell growth and differentiation, abnormal alterations of these signaling pathways result in malignancies. For example, the Src family of tyrosine kinases, whose members contain SH2 domains, play a role in both breast cancer [1] and osteoporosis [2], [3]. Their SH2 domains bind to pTyr-containing proteins, such as middle T antigen and various growth factor receptors [1], [4], and are therefore considered attractive targets for developing small-molecule inhibitors that would selectively disrupt these signaling processes. However, development of efficient small-molecule inhibitors has proven difficult, suggesting that a better understanding of the structural basis underlying phosphopeptide-SH2 domain interactions is required. To this end we have undertaken a computational and experimental study to systematically evaluate the role residues C-terminal to the pTyr anchor may play in the binding affinity and conformation of phosphopeptides when bound to the Src SH2 domain.

**Figure 1:**
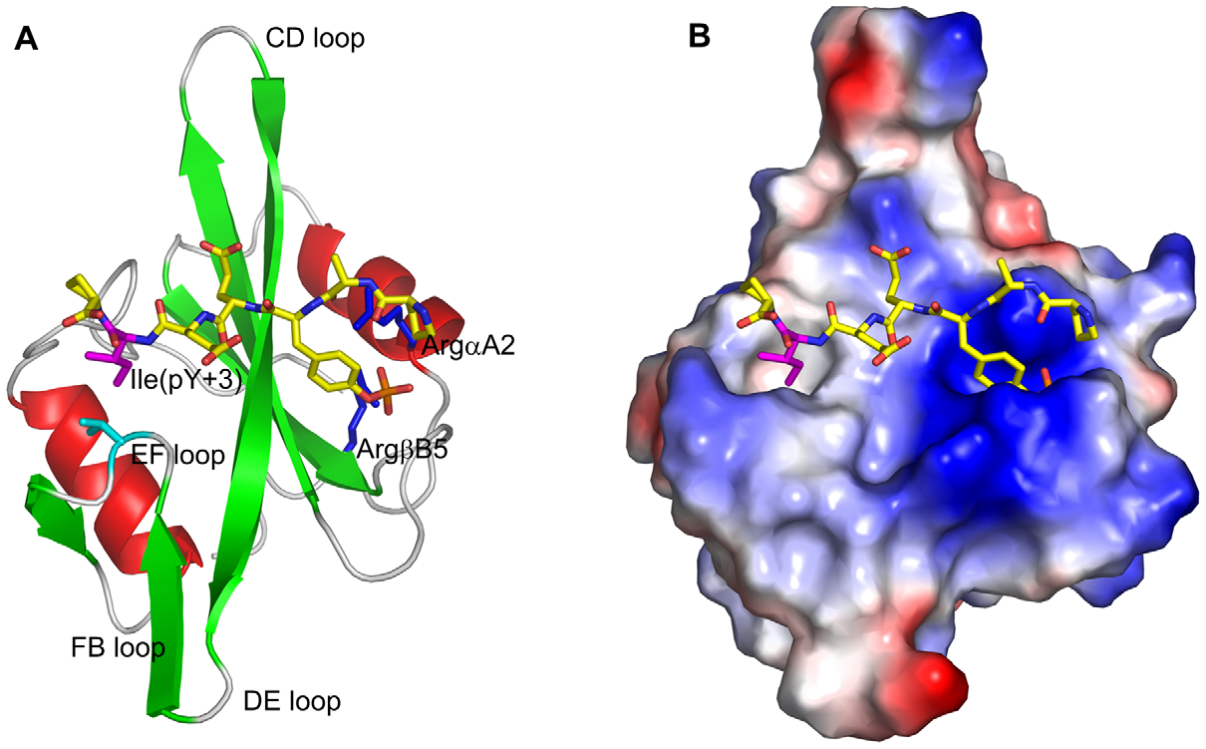
The Src SH2 domain complexed with pYEEI. (A) Cartoon representation of the complex. Residues ArgαA2 and ArgβB5 of the SH2 domain are in blue, residue Ile(pY+3) of the phosphopeptide is in magenta. (B) Electrostatic surface potential representation of the same complex. The phosphotyrosine sits in a highly electropositive hole, while residue Ile(pY+3) is inserted into the hydrophobic hole on the surface of the SH2 domain.

The Src SH2 domain binds with high affinity pTyr-Glu-Glu-Ile (pYEEI) [5]. The crystal [6], [7] and NMR [8] structures of this complex reveal that pYEEI adopts an extended conformation and that it forms its main interactions with the SH2 domain through the phosphorylated tyrosine residue, which binds into a positively charged pocket formed by two arginines (ArgαA2 and ArgβB5), and the isoleucine at the third position C-terminal to pTyr (pTyr+3), which binds into a hydrophobic pocket formed by the EF and FB loops (Figure 1). The contribution by the residue at position pTyr+3 has been demonstrated by the fact that substitution of smaller hydrophobic residues at this position results in decrease of binding affinity [9]. Based on these findings, the “two-pronged plug two-holed socket” model has been proposed, which postulates that binding of phosphopeptides to the Src SH2 domain is determined by phosphotyrosine and the hydrophobic residue at position pY+3. However, binding studies of conformationally constrained peptide analogs of pYEEI show that they have higher binding affinities than the unconstrained pYEEI [10], [11], suggesting that the “two-pronged plug two-holed socket” model may be an oversimplification and that any binding model must take into account the inherent flexibility of short peptides.

Since neither single-crystal X-ray diffraction methods, nor NMR methods have yielded a complete picture of the conformational diversity of phosphopeptides complexed with SH2 domains, we have turned to computational methods to explore the conformational trends of the bound phosphopeptides. Molecular simulations are well suited for this purpose; however, exhaustive sampling of the conformational space of even small peptides using fully solvated models, whether by Monte-Carlo methods or by molecular dynamics, is computationally expensive. A widely used computationally-efficient approach to this problem is simulated annealing, which is based on high-temperature sampling of selected degrees of freedom in implicit solvent [12]–[16].

Using this simulated annealing method in combination with fluorescence polarization techniques, we explore the conformational space of various phosphopeptides bound to the Src SH2 domain. We characterize the conformational landscape of the bound peptides by systematically examining the effect of different cooling rates, and we identify their conformational trends. We show that the nature of the residue at pTyr+3 plays an important, but not the key role in determining the binding affinity and the conformation of the bound peptide.

## Methods

### Phosphopeptide Assays

The relative binding affinity of phosphopeptides to the Src SH2 domain was tested by competition against a fluorescent probe using a fluorescence polarization (FP) binding assay [11], [17], [18]. FP was measured at 25°C in a disposable glass tube, using a Beacon 2000 luminescence spectrometer equipped with an FP apparatus. The excitation and emission wavelengths were set at 485 and 535 nm, respectively. The fluorescent probe was the fluorescein (Flc)-labeled phosphopeptide EPQ(pY)EEIPIYL(K-Flc). For the competition assay, final concentrations of 350 nM of GST-Src SH2 domain fusion protein, 50 nM fluorescent probe, HEPES buffer (20 mM, pH 7.3, 100 mM NaCl, 2 mM DTT, 0.1% BSA) and various concentrations (0–100 µM) of each competitor peptide were used. A blank control (with the Src SH2 domain but without a peptide) and a background control (without both the Src SH2 domain and the peptide) were used. The inhibition percentage *IP* of fluorescent probe binding to the Src SH2 domain by the sample was calculated by the following equation:
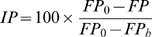
where *FP_0_* is the fluorescent polarization value of the blank control, *FP* is the fluorescent polarization value of the sample (peptide), and *FP_b_* is the fluorescent polarization value of the background control. The inhibition percentages of the various concentrations of the assayed peptides were plotted, and the IC_50_ value (the concentration that inhibits the binding of the fluorescent probe to the Src SH2 domain by 50%) was calculated.

### Molecular Dynamics Simulations

Sampling of the conformational space of the phosphopeptides complexed with the Src SH2 domain was carried out using a simulated annealing procedure in which a number of conformationally random replicas of the bound peptide are generated at high temperature, and then slowly cooled down. The conformation of the SH2 domain is assumed to remain unchanged; this assumption is based on single-crystal X-ray diffraction studies of both free and peptide-bound SH2 domains, which show that their structure is not affected by binding [6], [19]. At the end of the cooling process the main conformations present in a given replica population are identified by performing cluster analysis.

All molecular dynamics simulations were performed using the Sander-classic module in the AMBER6 package [20] with the *ff96* force field together with Verlet dynamics. (A basic description of the methods incorporated in AMBER is given elsewhere [21].)

The starting models for the simulated annealing procedure are either crystal structures of phosphopeptide-SH2 domain complexes, or – where such structures are not available – models built by mutating phosphopeptide residues from a closely related crystal structure. In both cases energy minimization preceded the start of the SA process.

The simulations were performed in a finite size, non-periodic system. Since exhaustive sampling of the full conformational space is beyond the reach of explicit-solvent simulations, the solvent effect is represented by a distance-dependent dielectric constant of the form ε = R*_ij_* (where R*_ij_* is the distance between particles *i* and *j*) [22]. Non-bonded interactions were calculated using a 12.0 Å cut-off radius. In order to prevent the SH2 domain from unfolding at high temperature, only the peptide residues C-terminal to the phosphotyrosine were allowed to move freely; harmonic restraints of 25 kcal/(mol*Å^2^) were applied to phosphotyrosine atoms, while the SH2 domain atoms were constrained to their positions in the starting models.

Partial charges for the phosphotyrosine were derived using the restrained electrostatic potential method (RESP) [23]; the ESP (electrostatic potential) input for RESP was generated using the package GAUSSIAN96 [24] with the 6-31G basis set [25]. The force field parameters of the phophotyrosine is supplied as Text S1. Since a distance-dependent dielectric does not screen electrostatic interactions between charged groups sufficiently well [26], all net charges were neutralized by using the protonated forms of Asp and Glu, and neutral forms of Lys and Arg, except for the peptide's phosophotyrosine and the for the SH2 domain residues ArgαA2 and ArgβB5, which form salt bridges to the phosphotyrosine (Figure 1); the N- and C-termini were neutralized by attaching acetyl (ACE) and N-methylamine (NME), respectively.

Before starting the SA protocol, the geometries of the starting models were optimized and bad interatomic contacts relieved by energy minimization consisting of 250 steps of steepest descent, followed by 750 steps of conjugate gradient minimization. Following the initial energy minimization, the SH2 domain complexed with the phosphotyrosine containing peptide was heated up to 3000K over three 10 ps long temperature steps, after which 50 samples were collected at this temperature at regular time intervals, varying between 2.5 and 10 ps. The time intervals were chosen so as to maximize both the conformational spread of the 50 copies, and their average CA-RMSD with respect to the starting model. Each structure was then cooled down using a logarithmic cooling protocol: at each cooling step *i*, the temperature *T* was chosen such that:
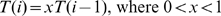
with *x* set to 0.8.

In order to characterize the energy landscape of the bound peptide, we tested various cooling rates, ranging from 10ps to 500ps per temperature step. This allowed us to identify structural trends of the peptide bound to the SH2 domain, as well as the rate at which these trends evolve, yielding a qualitative picture of the energy landscape.

The copies were cooled down to 300K, after which they underwent another round of energy minimization consisting of 200 steepest descent steps and 600 conjugate gradient steps. After all copies were cooled down and energy minimized, their CA-RMSDs from the initial structure were calculated and cluster analysis was performed by examining the joint rmsd-energy distribution, as well as using NMRCLUST (based on pair-wise RMSD) [27].

## Results

### Binding Affinities of Phosphopeptides

The IP_50_ of various phosphopeptides are presented in Table 1. The binding affinities of the various phosphopeptides to the Src SH2 domain only vary by one order of magnitude. The complexes of Src SH2 with peptides of type pYEEX (where X = I,L,V,A,G) have binding affinities correlated with the size of the residue at position pTyr+3, but the differences between the binding affinities are modest, as is also observed in calorimetric studes [9]. The contributions of the two glutamic acids at positions pTyr+1 and pTyr+2 to the binding affinity can be assessed by comparing the changes in IC_50_ effected by mutating these two residues, with the changes resulting from mutations of the residue at position pTyr+3. Substitution of Glu(pTyr+2) with Asn in pYEEI and pYEEV (to yield pYENI and pYENV, respectively), or of Ile(pTyr+3) with Val in pYEEI and pYENI causes a decrease in binding affinity by approximately 1.8 kJ/mol. Furthermore, replacing Glu(pTyr+1) with Val in pYENV causes a 3.3 kJ/mol decrease.

**Table 1:** Relative binding affinity of various phosphopeptides with the Src SH2 domain.

	IC_50_ (µM)	est. ΔG (kJ/mol)
pYEEI	0.5±0.2	−35.9
pYEEL	0.8±0.3	−35.2
pYEEV	1.1±0.1	−34.0
pYEEA	5.0±2.2	−30.2
pYEEG	5.7±2.1	−29.9
pYENI	1.0±0.1	−34.2
pYENV	2.1±0.7	−32.4
pYVNV	7.9±2.0	−29.1

IC_50_: concentration that reduces the binding of the fluorescein-labeled probe EPQ(pY)EEIPIYL(K-Flc) to the Src SH2 domain by 50%.

### pTyr-Glu-Glu-Ile-Pro

The starting model for this simulation was the crystal structure of the high-affinity (0.08–0.2 µM) [9], [28] complex of Src SH2 with pTyr-Glu-Glu-Ile-Pro (PDB code: 1SPS) [6]. The 50 structures generated at 3000K have an average CA-RMSD of 6±2 Å from the starting model, with values ranging between 2 and 12 Å, and 68% of the structures with RMSDs larger than 5 Å. The average potential energy is −280±13 kcal/mol, with values between −316 and −251 kcal/mol.

SA dynamics of this complex with various cooling schedules, which differ from each other by the time spent at each temperature level, shows that, even at relatively fast cooling schedules, most of the structures cluster close to the starting model.

**Figure 2:**
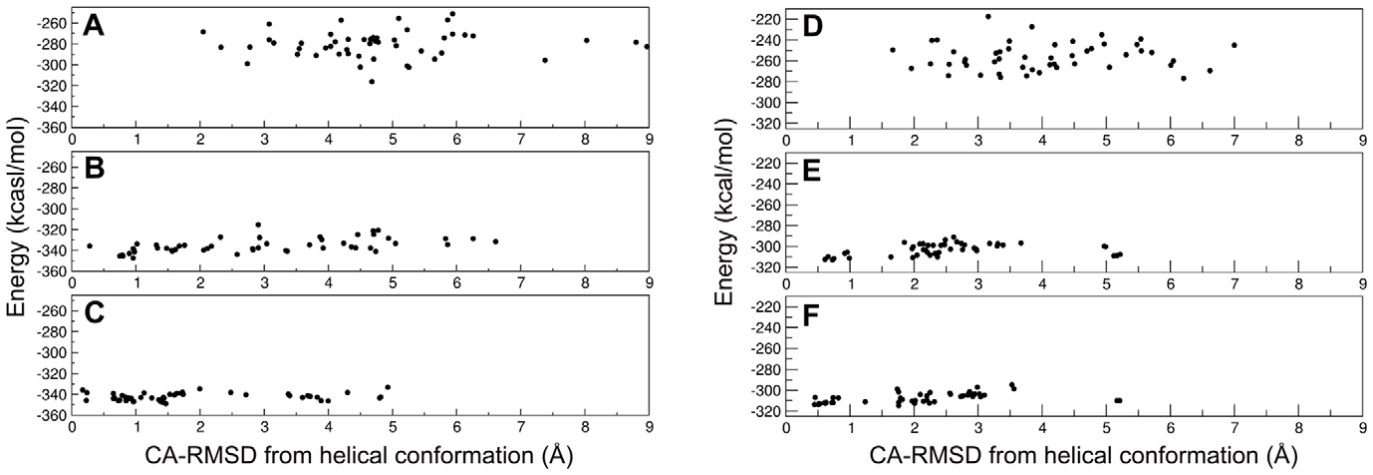
Spatial and energy distribution of the SA replicas of pYEEI and pYVPM complexed with Src SH2. (A and D) after energy minimization at 3000K; (B and E) after cooling with 50ps per temperature step; (C and F) after cooling with 500ps per temperature step.

**Figure 3:**
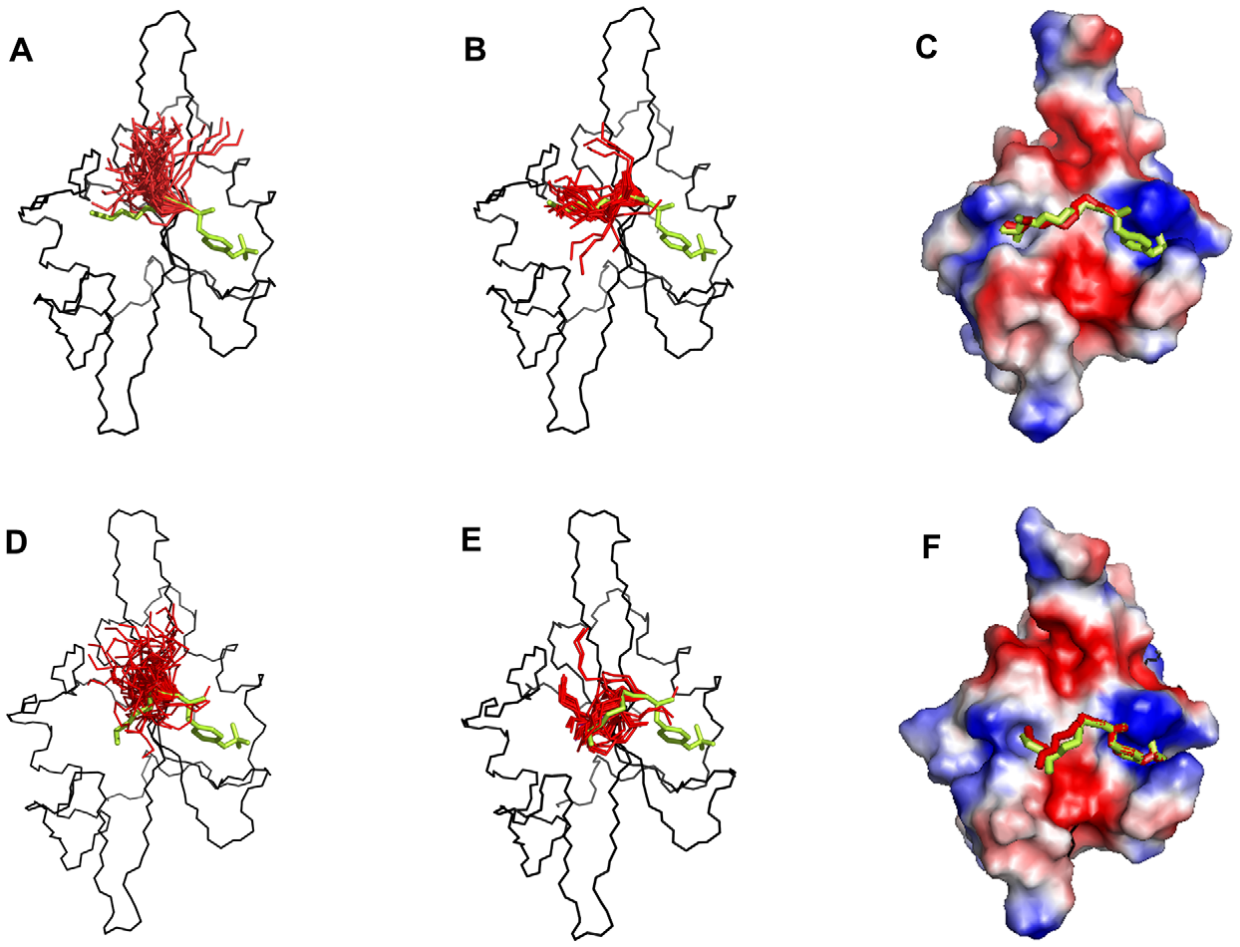
Conformational trends of pYEEI and pYVPM complexed with the Src SH2 domain. Backbone representation of the 50 computational replicas of pYEEI (A–C) and of pYVPM (D–F) superimposed on the crystal structures of these phosphopeptides in complex with the Src SH2 domain. (A and D) after energy minimization at 3000K; (B and E) after cooling with 500ps per temperature step. The backbone of the SH2 domain is in black, the SA replicas are in red. (C and F) Phosphopeptide backbone of the representative structure of the largest cluster after cooling with 500ps per temperature step, with side-chains of pTyr and of residue in position pTyr+3 (in red). The backbone of the crystal structure of the phosphopeptides and the side chains of the pTyr and residue pTyr+3 is in green. The SH2 domain is represented as an electrostatic potential surface. The representative structure of a cluster is the structure closest to the average structure of the cluster.

Cooling with 50 ps per temperature step results in an average CA-RMSD of 4±2 Å relative to the starting model and the average energy is −335±7 kcal/mol. At 500 ps per cooling step, the average CA-RMSD is 3±2 Å and the average energy is −342±3 kcal/mol. When considering only the three residues that form the specificity determining segment: Glu(pTyr+1)-Glu(pTyr+2)-Ile(pTyr+3), the average RMSD goes from 5±2 Å at 3000K (Figure 2A and 3A) to 3±2 Å after cooling at a rate of 50 ps/step, to 2±1 Å at 500ps/step, with 72% of the population (36 replicas out of 50) in a cluster with an average CA-RMSD of 1±0.5 Å (Figure 2B–C and 3B). Most of the structures in this cluster have the side chain of Ile(pTyr+3) inserted in the hydrophobic hole lined by residue ThrEF1 of the Src SH2 domain (Figure 3C). The remaining 14 copies have CA-RMSDs between 3 Å and 5 Å, with 8 of them in a tight cluster around 4 Å from the extended conformation, forming a distorted helix turn.

### pTyr-Val-Pro-Met-Leu

This complex is less specific, with a reported K_d_ of 5.5 µM [9]. In the crystal structure (PDB code: 1SHA) the phosphopeptide assumes a slightly bent extended conformation, with Met(pTyr+3) partially inserted in the hydrophobic pocket [29].

After cooling at a rate of 500 ps/step, the average CA-RMSD of the 50 copies of pYVPML relative to the crystal structure decreases from 6±2 Å to 3±2 Å, with 56% of the population in a cluster with a CA-RMSD of 1.5±0.5 Å. The CA-RMSD of the specificity determining segment Val(pTyr+1)-Pro(pTyr+2)-Met(pTyr+3) goes from 4.0±1 Å at 3000K, to 3±1 Å with a cooling rate of 50 ps/step, to 2±1 Å at 500 ps/step; 94% of the population lie in a broad cluster with an average CA-RMSD of 2±1 Å, and 28% of the population in a tight sub-cluster with a CA-RMSD of 0.6±0.1 Å (Figure 2D–F and 3D–F).

### The pTyr-Glu-Glu-(Hydrophobic) series

For the complexes of Src SH2 with peptides of type pYEEX (where X = I,L,V,A,G) the binding affinities correlated with the size of the residue at position pTyr+3 (Table 1). Since with the exception of pYEEI, no crystal structures are available for these complexes the starting models for this simulations were built from the crystal structure of the Src SH2-pYEEI complex (PDB code: 1SPS), in which the Ile(pTyr+3) was replaced by Leu, Val, Ala and Gly, respectively.

**Figure 4:**
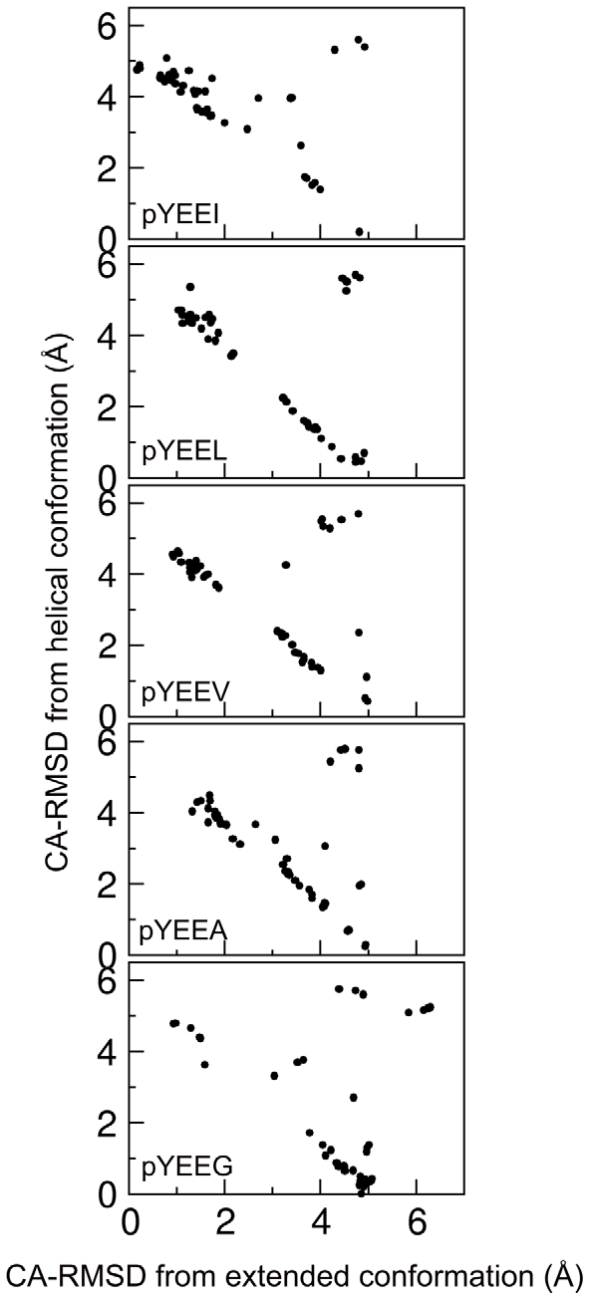
Spatial distribution of Simulated Annealing replicas of the pYEEX series between the extended and helical conformation after cooling at 500ps per step. From top to bottom: pYEEI, pYEEL, pYEEV, pYEEA, pYEEG.

**Figure 5:**
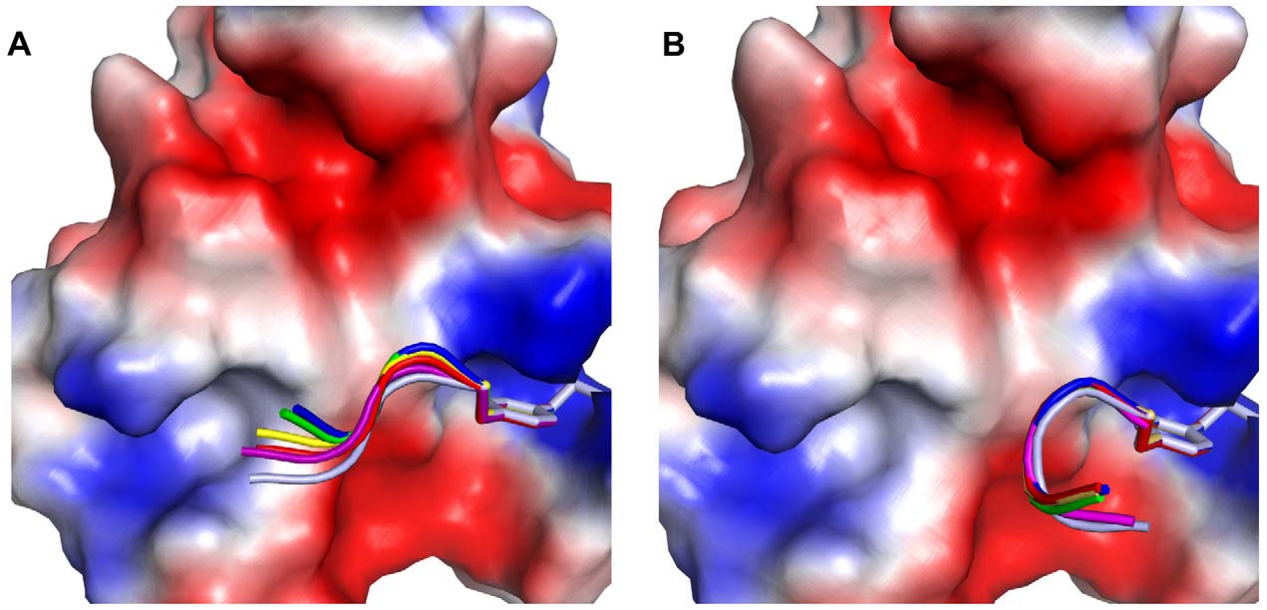
Conformational trends of the pYEEX series of peptides complexed with the Src SH2 domain. (A) Cartoons of representative structures of the “extended” conformation cluster of the pYEEX series superimposed on the structure of pYEEI complexed with the Src SH2 domain; (B) Representative structures of the “helical” conformation cluster of pYEEX series, superimposed on an ideal helix. The reference conformations for extended and helical clusters are in magenta, pYEEI, red, pYEEL, yellow, pYEEV, green, pYEEA blue, pYEEG, light blue.

For all five phosphopeptides, SA with 500ps/step results in 80% of the population located in two clusters, indicating two main tendencies for the phosphopeptide conformation (Figure 4). One cluster is close to the extended conformation observed in the pYEEI-Src SH2 complex (CA-RMSD = 1–2 Å); the other cluster, with CA-RMSD = 4–5 Å is close to a helical conformation (Figures 4 and 5). As the size of the side chain of pTyr+3 residue decreases, so does the population of the first cluster, and the deviation from the extended conformation increases (CA-RMSD of 1.5 Å for pYEEL and pYEEV, and 2 Å for pYEEA). The population of the second cluster increases and its deviation from the ideal helical conformation decreases. The extreme cases are pYEEI and pYEEG, where more than 70% of the population is in the extended and helical conformation, respectively. The deviation from the extended conformation in the first cluster is caused by a bend at residue +3 in the direction of the CD loop and away from the hydrophobic pocket of the SH2 domain (Figure 5). A third, very small cluster (comprising 6 to 12% of the population) with CA-RMSD of 5 Å from the extended conformation and 6 Å from the helical one can be observed in all five complexes; it corresponds to a conformation in which Glu(pTyr+1) is rotated by about 180° around the N-CA bond, resulting in the side chain of Glu(pTyr+1) pointing towards the DE loop and in the main chain of the peptide bending by almost 90° towards the CD loop.

### pTyr-Glu-Asn-Ile and pTyr-Val-Asn-Val-Ala

These two peptides have lower binding affinities to Src SH2, with IC_50_ of 1.0 µM for pYENI and 7.9 µM for pYVNV (Table 1). pYVNV binds tightly to the Grb2 SH2 domain, as well as to a mutant of Src SH2 where residue ThrEF1 is replaced by Trp [28]. In those complexes the peptide assumes a β-turn conformation [19], [30], with residue Asn(pTyr+2) forming hydrogen bonds with the SH2 domain main chain's peptide groups, namely the NH of LysβD6 and the carbonyl of LysβD6 and IleβE4.

The starting model for the simulation of both pYENI and pYVNV complexes was the crystal structure of the pYEEIP-Src SH2 complex [6]; for pYENI, Glu(pTyr+2) was mutated to Asn, whereas for pYVNV the three residues C-terminal to pTyr, Glu(pTyr+1), Glu(pTyr+2) and Ile(pTyr+3), where mutated to Val, Asn and Val, respectively.

**Figure 6:**
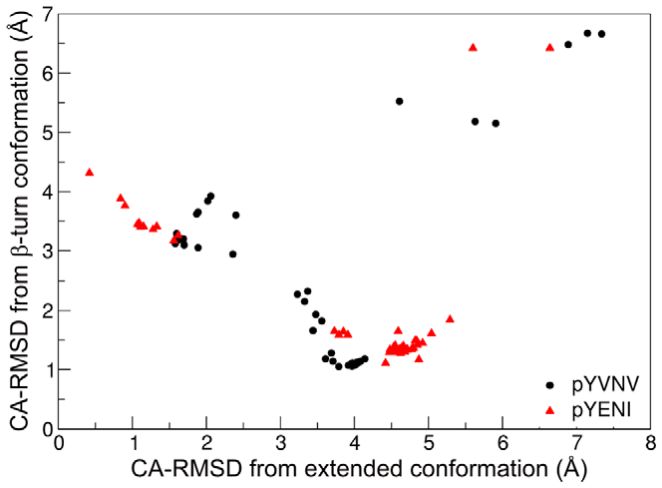
Spatial distribution of the replicas between extended and β-turn conformation for pYVNV *(black)* and pYENI (*red)*, after cooling with 500ps per temperature step.

**Figure 7:**
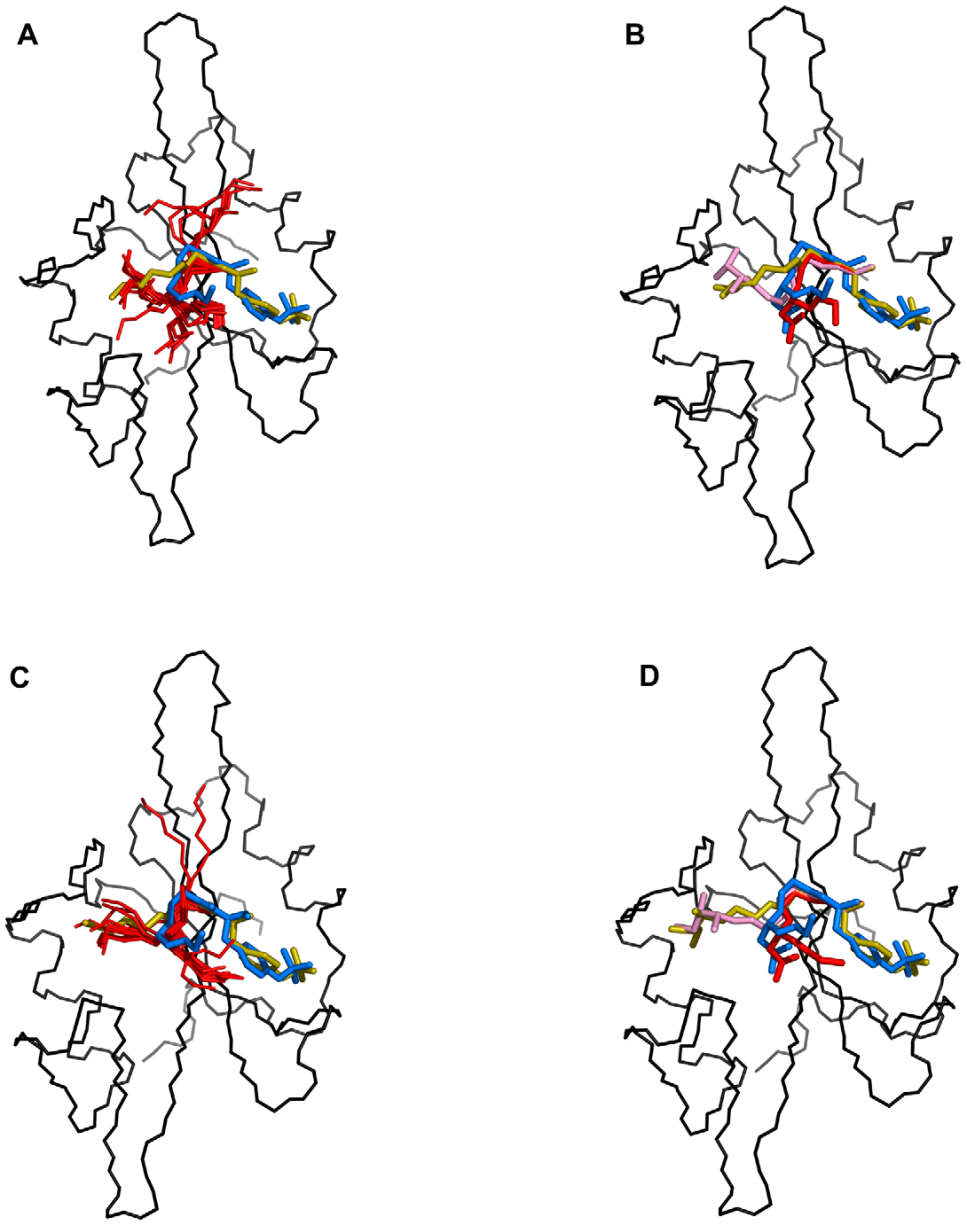
Conformational trends of the pYVNV and pYENI complexed with the Src SH2 domain. *(left)* Backbone representations of the 50 replicas (red) superimposed on the crystal structure of the pYVNV-ThrEF1Trp Src SH2 domain complex and the modeled extended conformation of pYVNV for (A) pYVNV and (C) pYENI. *(right)* Backbone of the representative structure of the main cluster (red) and minor cluster (pink) with the side-chain of residue Asn(pTyr+2) for (B) pYVNV and (D) pYENI. Backbone of SH2 domain in black, modeled extended conformation of pYVNV in yellow, β-turn conformation of pYVNV in blue.

**Figure 8:**
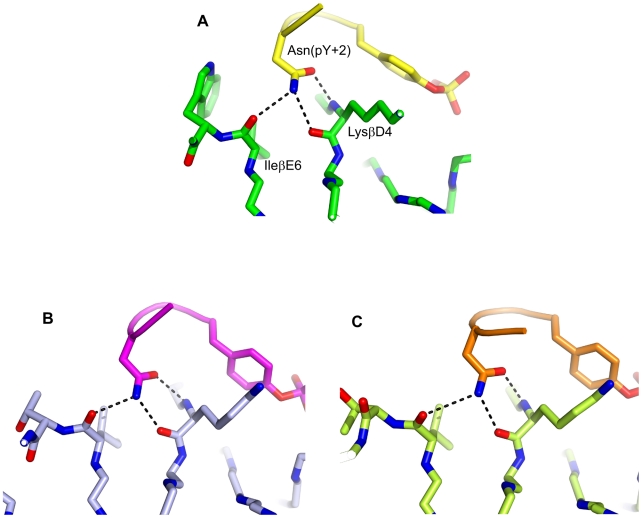
Hydrogen bonds formed by Asn(pY+2) in pYVNV and pYENI complexed with SH2 domains. (A) pYVNV complexed with the ThrEF1Trp mutant of Src SH2; (B) pYVNV complexed with wt Src SH2 (representative of the major cluster); (C) pYENI complexed with wt Src SH2 (representative of the major cluster). The phosphopeptide backbone is represented as a cartoon.

In both complexes, SA results in major clusters with CA-RMSDs of 4.0–4.5 Å from the extended conformation (Figure 6), which corresponds to the β-turn conformation observed in the structure of pYVNV complexed with Grb2 SH2 or with the ThrEF1Trp mutant of Src SH2 (Figure 7). In the case of pYVNV, 50% of the population is in the β-turn conformation, 25% of the population forms a slightly broader cluster with a CA-RMSD of 2 Å from the extended conformation, and another 12% lie in a cluster about 6 Å from both the extended and the β-turn conformation, corresponding to a conformation in which the peptide forms a roughly 90° bend towards the CD loop. In the pYENI complex, 60% of the population is in the β-turn conformation and 30% in the extended conformation with Ile(pTyr+3) inserted in the hydrophobic pocket of the SH2 domain, as observed in the high-affinity complex of pYEEI. In both complexes, residue Asn(pTyr+2) forms the same polar contacts with the main-chain amino and carbonyl groups observed in the crystal structures of pYVNV complexed with the Grb2 SH2 domain or with the mutant ThrEF1Trp of the Src SH2 domain (Figure 8).

## Discussion

The results obtained using our simulated annealing protocol are consistent with those obtained by other theoretical and experimental studies. Specifically, the deviations from the extended conformation observed in the SH2-bound peptides pYEEA and pYEEG are similar to those reported in a more sophisticated, parallel tempering dynamics study of Ala and Gly mutants of pYEEI bound to Src SH2 with implicit solvent and a different energy function [31]. Likewise, the conformation predicted by our computational technique for the pYXN fragment of pYVNV and pYENI complexed with Src SH2 agree with the one obtained using a Monte Carlo search [32]. Most significantly, the conformational trends identified by the simulated annealing method for the pYEEI and pYVPM peptides bound with, respectively, high and low affinity to the Src SH2 domain are in very good agreement with the conformations observed in the crystallographic structures of these complexes [6], [29]. These results show that in spite of its approximations, i.e.: use of implicit solvent, neutralization of local charges, constraining the structure of the SH2 domain, the simulated annealing approach, as implemented in this work, yields reliable results in identifying the conformational trends of phosphopeptides complexed with SH2 domains. Using various cooling rates allowed us to monitor the rate at which the population clusters are developing, which, in turn, is indicative of the heterogeneity of the energy landscape for this system.

The results of the fluorescence polarization assays suggest that the two glutamic acids at positions pTyr+1 and pTyr+2 provide a significant contribution to the binding energy through their electrostatic interactions with positively charged residues on the surface of the Src SH2 domain, and that all three residues C-terminal to the pTyr are equally important in determining the binding affinity.

In all complexes with the Src SH2 domain, the fragments of type pYEEX (X = I,L,V,A,G) adopt two main conformations: one fully or partially extended, the other one partially helical. The presence of a helical conformation for the pYEEX fragment is entirely consistent with the strong helical propensity of Glu residues [33], [34]. As the size of the residue at position pTyr+3 decreases, the tendency towards the extended conformation decreases too, while the number of replicas in a helical conformation increases. However, the correlation between the size of residue pTyr+3 and the structural trends displayed by the corresponding phosphopeptide is not straightforward: as one can see in Figure 4, in the case of both pYEEL and pYEEV the populations of the extended and helical conformations are very close (50% and 40% of the population, respectively), even though Val is smaller than Leu. This can be explained by the fact that while Leu is a very strong helix former, it also interacts strongly with the hydrophobic hole of the Src SH2 domain, whereas Val has only weak helical propensity, but interacts only weakly with the hydrophobic hole; in both cases, therefore, the two opposing tendencies balance each other, resulting in roughly equal populations. At the same time, the structural trends of pYEEL and pYEEI are strikingly different, even though Leu and Ile are very close in size: in the case of pYEEI 72% of the population is in the extended conformation and only 16% adopts the helical conformation. This, too, can be explained by the difference in helical propensities between Leu and Ile: while both amino-acids interact in a similar manner with the Src SH2 hydrophobic hole, Ile has a much weaker helical propensity than Leu. Therefore, the conformation of the pYEEX fragment in the complex with the Src SH2 domain appears to be the result of the competition between the additional contribution of residue pTyr+3 to the helical propensity of the two glutamic acid residues at the first and second position after the phosphotyrosine, on the one hand, and its interaction with the hydrophobic hole of Src SH2 domain, on the other hand.

The fact that when pYVNV is bound to the Src SH2 domain, it adopts mainly a β-turn conformation with Asn(pTyr+2) forming non-specific polar contacts with the protein, confirms the prediction that in addition to pYEEI the Src SH2 domain is also capable of binding peptides of the type pYXNX [32]. According to this prediction, these peptides should assume a β-turn conformation, except in the case when the residue at position pTyr+3 engage in strong interactions with the hydrophobic hole on the surface of the Src SH2 domain. Therefore, one would expect the pYENI fragment to adopt mainly the extended conformation, because of the presence of Ile at position pTyr+3. However, the population of the β-turn conformation of pYENI bound to Src SH2 is higher than that of the extended conformation. This suggests that the presence of Asn at position pTyr+2 can compete strongly with the role of Ile at position pTyr+3 as both an affinity and structure determinant.

In conclusion, we show that all three residues C-terminal to the phosphotyrosine play an important role in determining both the binding affinity of phosphopeptides complexed with the Src SH2 domain, as well as their conformation. Significantly, the conformational trends of the bound phosphopeptides are a result not only of the phosphopeptide-SH2 domain interactions, but also of the intra-phosphopeptide interactions. These observations can support the design of better, more specific inhibitors of the Src SH2 domain.

## Supporting Information

Text S1 Phosphotyrosine force field parameters.
(0.03 MB DOC)
