# Supplementary material for: Conformational Determinants of Phosphotyrosine Peptides Complexed with the Src SH2 Domain
Source: PLoS One. 2010 Jun 21;5(6):e11215. doi: 10.1371/journal.pone.0011215 (PMC2888578; doi:10.1371/journal.pone.0011215)
Supplement: Text S1 — Phosphotyrosine force field parameters. (0.03 MB DOC) [file pone.0011215.s001.doc]

PHOSPHOTYROSINE

pty.db96

PTY INT 1

CORR OMIT DU BEG

1 DUMM DU M 0 -1 -2 0.000 0.000 0.000 0.00

2 DUMM DU M 1 0 -1 1.449 0.000 0.000 0.00

3 DUMM DU M 2 1 0 1.522 111.100 0.000 0.00

4 N N M 3 2 1 1.335 116.600 180.000 -0.53

5 H H E 4 3 2 1.010 119.800 0.000 0.32

6 CA CT M 4 3 2 1.449 121.900 180.000 0.19

7 HA H1 E 6 4 3 1.090 109.500 300.000 0.06

8 CB CT 3 6 4 3 1.525 111.100 60.000 -0.32

9 HB2 HC E 8 6 4 1.090 109.500 300.000 0.07

10 HB3 HC E 8 6 4 1.090 109.500 60.000 0.07

11 CG CA S 8 6 4 1.510 109.470 180.000 0.03

12 CD1 CA B 11 8 6 1.390 120.000 180.000 -0.17

13 HD1 HA E 12 11 8 1.080 119.470 0.000 0.11

14 CE1 CA B 12 11 8 1.380 121.800 180.000 -0.44

15 HE1 HA E 14 12 11 1.070 120.760 180.000 0.11

16 CZ C B 14 12 11 1.410 121.230 0.000 0.73

17 OH OS S 16 14 12 1.290 124.710 180.000 -0.61

18 P P 3 17 16 14 1.850 126.000 -11.860 1.38

19 O1P O2 E 18 17 16 1.496 100.160 57.820 -0.92

20 O2P O2 E 18 17 16 1.496 100.160 -61.530 -0.92

21 O3P O2 E 18 17 16 1.496 100.160 177.950 -0.92

22 CE2 CA B 16 14 12 1.410 116.420 0.000 -0.44

23 HE2 HA E 22 16 14 1.070 117.320 180.000 0.19

24 CD2 CA S 22 16 14 1.380 121.910 0.000 -0.17

25 HD2 HA E 24 22 16 1.080 121.230 180.000 0.19

26 C C M 6 4 3 1.522 111.100 180.000 0.59

27 O O E 26 6 4 1.229 120.500 0.000 -0.57

LOOP

CG CD2

IMPROPER

-M CA N H

CA +M C O

CG CE2 CD2 HD2
